# Improving rigid fiberoptic intubation: a comparison of the Bonfils Intubating Fiberscope™ with a novel modification

**DOI:** 10.1186/1471-227X-10-11

**Published:** 2010-05-27

**Authors:** Ben H Boedeker, Mary A Barak-Bernhagen, David J Miller, Thomas A Nicholas, Andrew Linnaus, WB Murray

**Affiliations:** 1 University of Nebraska Medical Center, Omaha, NE, USA; 2 Omaha VA Medical Center, Omaha, NE, USA; 3 Clinical Simulation Center, Penn State College of Medicine, Hershey, PA, USA

## Abstract

**Background:**

The Bonfils intubating fiberscope has a limited upward tip angle of 40° and requires retromolar entry into the hypopharynx. These factors may make its use less desirable when managing the difficult airway because most anesthesia providers are well versed in midline oral intubation rather than the lateral retromolar approach. The *Center for Advanced Technology and Telemedicine *at the University of Nebraska Medical Center has developed a novel fiberscope with a more anterior 60° curve to allow for easier midline insertion and intubation. The objective of this work was to evaluate the novel fiberscope, in comparison to the Bonfils intubating fiberscope, in terms of use and function in difficult airway intubation.

**Methods:**

Twenty-two anesthesia providers participated in simulated intubations of a difficult airway mannequin to compare the Bonfils intubating fiberscope with the novel curved Boedeker intubating fiberscope. The intubations were assessed based upon the following variables: recorded Cormack Lehane airway scores, requests for cricoid pressure, time to intubation, number of intubation attempts and success or failure of the procedure.

**Results:**

Participants using the Bonfils fiberscope recorded an average Cormack Lehane (CL) airway score of 1.67 ± 1.02 (median = 1); with the novel fiberscope, the recorded average airway grade improved to 1.18 ± 0.50 (median = 1). The difference in airway scores was not statistically significant (p = 0.34; Fishers Exact Test comparing CL grades 1&2 vs. 3&4). There was, however, a statistically significant difference in intubation success rates between the two devices. With the Bonfils fiberscope, 68% (15/22) of participants were successful in intubation compared to a 100% success rate in intubation with the novel fiberscope (22/22) (p = 0.008). After the intubation trial, the majority of participants (95%) indicated a preference for the novel fiberscope (n = 20).

**Conclusions:**

With this data, we can infer that the novel fiberscope curvature appears to improve or maintain the quality of an intubation attempt (airway score, cricoid pressure requirement, intubation time, number of attempts, placement success). The data indicate that the novel fiberscope offers a superior intubation experience to currently available best practices. The instrument was well received and would be welcomed by most study participants should the device become clinically available in the future.

## Background

Management of the difficult airway is a considerable challenge for anesthesia providers and is the major cause of morbidity and mortality. When confronted with a patient who has a predicted difficult airway (difficulty in opening of the mouth, lack of mobility of the atlanto-occipital joint, inability to assume the sniffing position), intubation may be extremely formidable. In cases such as these, it may be more advantageous to secure the airway while the patient is still awake. An airway device that allows for intubation without the need of a straight line of sight while lifting and navigating through airway structures would be beneficial.

Multiple types of devices have been developed to avoid having a straight line of sight. A common methodology is to move the point of sight (using a miniature camera) to the tip of a standard (or modified) rigid laryngoscope (e.g. the various forms of videolaryngoscope, including the Airtraq). The endotracheal tube is then passed separately next to the device. The early passage is essentially blind, until the tip of the endotracheal tube enters the view of the camera. The rigid Bonfils Intubating Fiberscope has the endotracheal tube mounted (threaded) on the device, thereby acting as a fiberscope. The pathway is always in view, and the operator's second hand is free to perform other tasks.

Prior studies have demonstrated the usefulness of the Bonfils Intubating Fiberscope during difficult intubation [1-6] as well as in awake intubation of the difficult airway [1]. Some of the Bonfils Intubating Fiberscope's characteristics, however, may be less than desirable when applied to a patient with a difficult airway. For example, the Bonfils Intubating Fiberscope has a moderate curvature (40°), and therefore requires a retromolar or lateral entry into the hypopharynx. This lateral entry (at an angle across the tongue) is unfamiliar to most anesthesia practitioners, and has another added learning curve to using this instrument. Most anesthesia providers are more accustomed with a midline approach for oral intubations.

**Figure 1:**
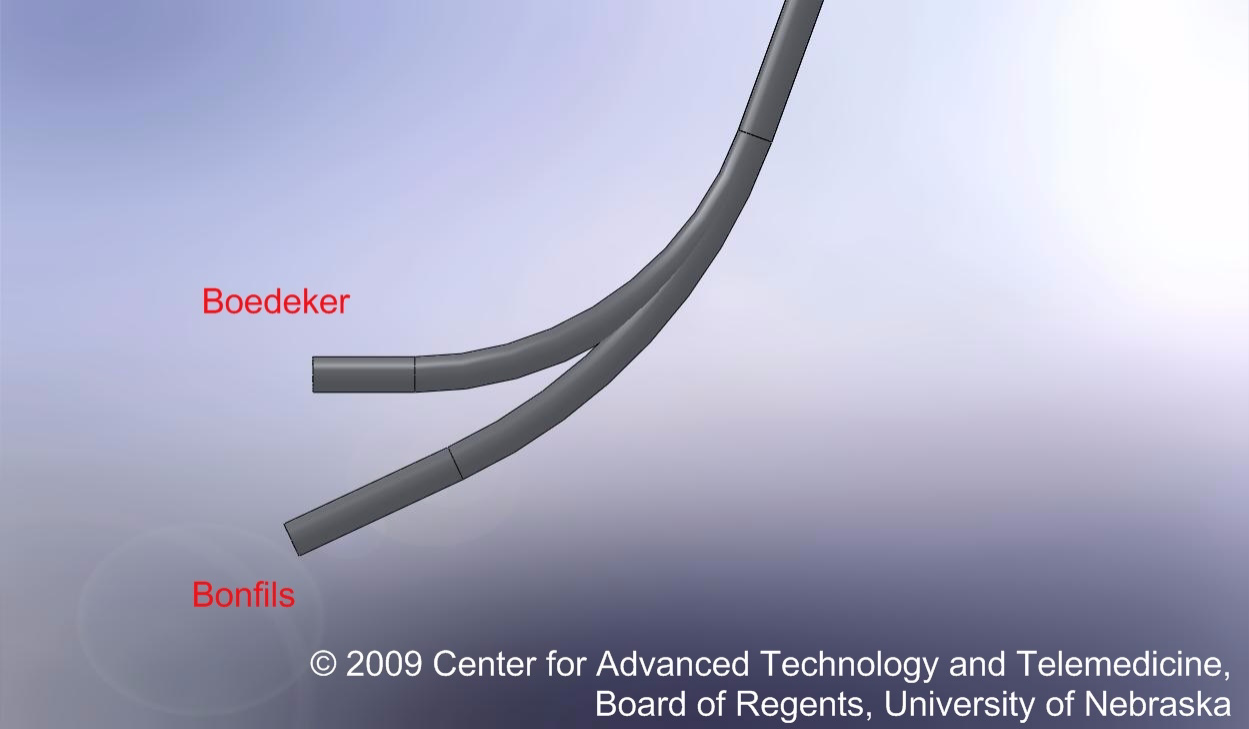
**Comparison of the curvature of the Boedeker vs. Bonfils intubation fiberscopes**. The Boedeker fiberscope has a greater curvature of 60°. This more anterior curve, compared to the Bonfils Intubation Fiberscope (40 degrees), allows it to line up more easily with the larynx and vocal cords. It also allows insertion in the midline.

**Figure 2:**
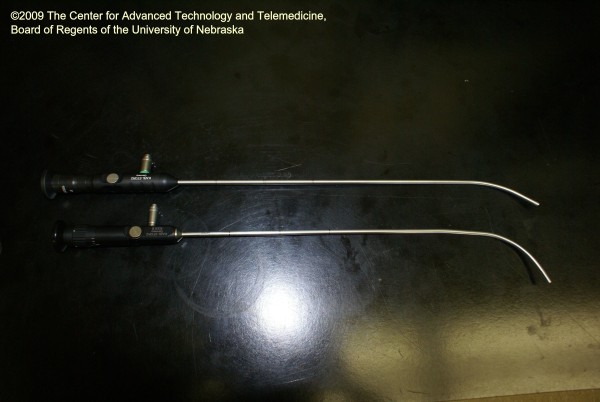
**The Bonfils and Boedeker fiberscopes**. Photo shows the Bonfils (top) and Boedeker (bottom) fiberscopes.

Based on the above design and use limitations, the Boedeker intubating fiberscope (Figures 1 and 2) was fabricated by altering a 15 Fr Bonfils Intubating Fiberscope (KARL STORZ Endoscopy, El Segundo, CA) with a modified angle of 60°. The novel curvature of this fiberscope allows the provider to use the more familiar midline approach for intubation. The objective of the current study is to compare the newly designed Boedeker intubating fiberscope with the Bonfils Intubating Fiberscope in the intubation of a simulated difficult airway in terms of use and functional characteristics.

## Methods

Following IRB approval, anesthesia providers (n = 22) including anesthesia attending physicians and residents, and Certified Registered Nurse Anesthetists (including one student CRNA) at the University of Nebraska Medical Center and Omaha VAMC, Omaha, NE participated in intubation of a Laerdal Deluxe Difficult Airway Trainer™ (Laerdal Medical Corporation, Wappingers Falls, NY) with the tongue inflated to simulate a difficult airway [7]. The providers completed a pre-experience questionnaire assessing prior experience with awake intubation, and their level of training.

Prior to the exercise, the instructor demonstrated the use of both fiberscopes. The participants were then observed during their intubation attempts alternatively using the Bonfils and Boedeker intubating fiberscopes (randomized to eliminate learning effects) (Figures 1 and 2). During the study, the following variables were collected: recorded Cormack Lehane (CL) airway score, the time to intubation, the number of intubation attempts, the success/failure of the intubation, and whether or not cricoid pressure was requested by the intubator. The observed view of the glottic opening was graded by the participant using the Cormack Lehane (CL view) grading scale (where Grade I = full view of the glottic opening; Grade II = posterior portion of glottic opening is visible; Grade III = only the tip of the epiglottis is visible; Grade IV = only the soft tissue is visible). Following their intubation experience, the providers completed a questionnaire expressing their device preference and whether or not they had previously heard about retromolar intubation.

### Statistics

The significance of non-parametric data (e.g. categorical data such as the airway view grading (CL grades 1&2 vs. 3&4), success/failure rate and rate of cricoid pressure requests were calculated using a Fisher's Exact Test. For the observed airway views, Cormack Lehane grades "1&2" were combined as "good views", and grades "3&4" were combined as "poor views." Values for the airway are reported as median. Timing comparisons and number of intubation attempts were recorded as means ± standard deviation and calculated using a Paired t-Test. A p-value < 0.05 was considered significant.

## Results

Study participants consisted of one Student Registered Nurse Anesthetist, 5 Certified Registered Nurse Anesthetists and 16 MDs (8 residents and 8 attending), all of whom were anesthesia practitioners. At the time the study was conducted, the level of experience of the anesthesia practitioners in awake intubation ranged from having no experience to having 50+ (one subject had no experience; six had experience in less than 20 awake intubations; fifteen had experience in 20 or more awake intubations) (see Table 1). Twenty-three percent (5/22) of the participants had previously heard of the retromolar intubation technique compared to 77% (17/22) that had not.

**Table 1:** Previous experience of study participants in awake intubation attempts

Experience In Awake Intubations	0	<10	<15	<20	>20	>30	>50
% of Study Participants n = 22	1	4	1	1	7	6	2

In comparing the recorded median Cormack Lehane airway view scores between the two devices (both medians = 1), there is no significant difference. A breakdown of the recorded Cormack Lehane airway scores is shown in Table 2. The data indicates that with the Boedeker fiberscope, 95% recorded a good view (Grades 1&2) and 5% recorded a poor view (Grades 3&4). With the Bonfils fiberscope, 81% recorded a good view (Grades 1&2) vs. 19% recording a poor view (Grades 3&4).

**Table 2:** Airway scores, number of intubation attempts and average time to intubation using the Boedeker versus the Bonfils Fiberscope

Test	Boedeker Fiberscope	Bonfils Fiberscope	P value
Cormack Lehane Airway Score Median (Grade I, II, III, IV)	n = 21 I (18,2,1,0)	n = 21 I (13,4,2,2)	0.34

Average # of intubation attempts	n = 22 1.09 ± 0.43	n = 22 1.09 ± 0.29	1.00

Average Time to Intubation (seconds)	n = 19 17.66 ± 10.84	n = 18 21.81 ± 11.65	0.27

The data shows that there was essentially no difference in average times to intubation (p = 0.27) or in the average number of intubation attempts (p = 1.00; unpaired t-test) between the two devices (Table 2).

**Figure 3:**
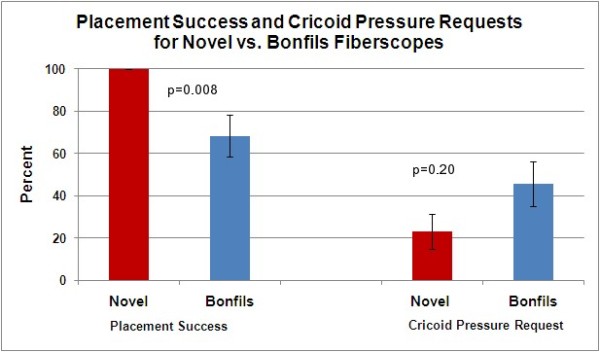
**Success of intubation rates and rates of requests for cricoid pressure during intubation attempts**.

The data expresses a statistically significant difference in intubation success rates between the two devices. In intubation with the Bonfils fiberscope, 68% (15/22) of participants were successful compared to a 100% success rate in intubation with the novel fiberscope (22/22) (p = 0.008) (Figure 3).

For the requests for cricoids pressure, when using the Boedeker fiberscope, 23% (5/22) of participants requested cricoid pressure as compared to 45% (10/22) of participants requesting cricoid pressure with the Bonfils fiberscope. Although there is a trend evident, the difference between the two devices is not statistically significant (p = 0.20) (Figure 3).

A majority (95%; 19/20) of the study participants preferred the Boedeker fiberscope when asked which device they had a preference for (n = 20). Comments by the participants were invited and collected and included the following: "Novel curve was easier to maneuver"; "Didn't like curve of Bonfils"; "Bonfils harder to manipulate".

## Discussion

As previously established [5], our study confirms that both rigid fiberscopes provide good views of the difficult airway (as reflected in the low CL airway view scores-median view score = 1 for each). It is interesting to note, however, that with the Boedeker fiberscope, there is a trend showing more observed airway scores with a low (or good) airway view score of 1 or 2 (95% or 20/21) than that seen with the Bonfils fiberscope (81% or 17/21). This difference is not statistically significant. (p = 0.34).

Due to the widespread popularity of the Bonfils intubating fiberscope, it stands to reason that users would take few tries to achieve a successful intubation. The interesting point to notice from Table 2 is the fact that the number of intubation attempts and the times to intubation were not statistically significantly different for both fiberscopes, although the Bonfils fiberscope was inserted retromolar and the Boedeker fiberscope was inserted midline.

The most dramatic difference between the two instruments was observed in the successful intubation rates (as shown in Figure 3). The data collected indicated that using the Boedeker fiberscope lead to a significantly higher intubation success rate (100%) than with the Bonfils fiberscope (68%) (p = 0.008).

With respect to requests for cricoid pressure during the intubation procedure, fewer requests (23%) were recorded when using the Boedeker fiberscope compared to 45% with the Bonfils. This difference is not statistically significant (p = 0.20) most likely due to the small sample size, but this trend is interesting.

Two limitations to this study were the small sample size and the varied experience of the study participants in awake intubation. There was a very large standard deviation among the times to intubation. This is most likely due to the varied experience of the operators. For the most part, since the scenarios were randomized to eliminate any learning effects, if the users were inexperienced, they were slow to intubate in both the scenarios, leading to a wide range of intubating times. It is interesting to note that the values in Table 2 show that the lowest time to intubation was for the novel fiberscope. Another possible contributing factor to our large standard deviation would be the lack of training in using the rigid intubating fiberoptic devices. A majority of the participants (77%) had no experience with the Bonfils or the retro-molar technique.

For the most statistical power, one would like all participants to have minimal variation, and the standard deviation would be at the lowest range; however, the generalization to a different population is much less robust/applicable. Given a group such as ours, the study is more applicable to the population found in a typical medical institution. The wide standard deviation is an indication that trainees and novices to these techniques will have a wide range of training needs. Giving everyone a "time based" learning experience would not suffice.

Prior studies have identified the learning curve associated with the Bonfils [3,6,8]. In these prior (published) studies, it was determined that 20 training intubations needed to occur before the operator would be considered to be proficient with a non-difficult airway. The studies also identified that 50 intubations must occur before an intubator is proficient with "difficult airways." Certainly, the investigators will consider addressing these training requirements and selecting a larger sample size when future intubating fiberscope studies are undertaken.

The authors believe that the new device shows improvement in the intubation experience; however, due to the large standard deviations present in this data, the sample size should be increased to fully investigate the significance of the claims. The novel instrument was also well accepted among study participants indicating that, if available, most users would prefer using this novel fiberscope over the Bonfils when warranted for difficult airway intubation. Many of the participants in the study commented that it would be easier to tell which was the better solution (Boedeker vs. Bonfils fiberscope) in a real OR setting. To that end, the device is being taken through the FDA approval process so that it can be used on humans in the OR.

## Conclusions

Based on the data in our study, the novel curvature of the Boedeker fiberscope appears to improve and/or maintain the quality of an intubation attempt (in respect to airway score, cricoid pressure requirement, intubation time, number of attempts, placement success, and operator preference). In this study, the difference between the two devices with respect to the intubation success rates is statistically significant with the Boedeker fiberscope providing a 100% success rate versus 68% with the Bonfils. Our data has shown that the Boedeker fiberscope offers a superior intubation experience to that of the Bonfils fiberscope. As the new device was well received by the study participants, it is believed by the authors that many users would choose to include this device on their standard airway carts should it become clinically available in the future.

## Competing interests

The University of Nebraska Board of Regents holds all of the intellectual properties associated with this project. The authors declare that they have no competing interests.

## Authors' contributions

BHB conceptualized the device and its design. BHB designed the study protocol, the questionnaire, performed the testing and data collection and contributed to the manuscript preparation. MAB assisted in data analysis and manuscript development. DJM assisted in the device engineering, device testing, data collection and analysis, and manuscript preparation. TAN assisted in data collection and manuscript development. AL assisted in data collection and data analysis. WBM assisted in manuscript development and statistical analysis. All of the authors have read and approve of the final manuscript.

## Pre-publication history

The pre-publication history for this paper can be accessed here:

http://www.biomedcentral.com/1471-227X/10/11/prepub
